# Carboxylated Graphene Oxide as a Nanocarrier for Drug Delivery of Quercetin as an Effective Anticancer Agent

**DOI:** 10.52547/ibj.3598

**Published:** 2022-08-13

**Authors:** Fatemeh Yaghoubi, Najmeh Sadat Hosseini Motlagh, Ali Moradi, Fateme Haghiralsadat

**Affiliations:** 1 Department of Clinical Biochemistry, School of Medicine, Shahid Sadoughi University of Medical Sciences, Yazd, Iran;; 2 Medical Nanotechnology and Tissue Engineering Research Center, Yazd Reproductive Sciences Institute, Shahid Sadoughi University of Medical Sciences, Yazd, Iran;; 3 Dapartment of Biomedical Engineering, Meybod University, Meybod, Iran;; 4 Department of Advanced Medical Sciences and Technologies, School of Paramedicine, Shahid Sadoughi University of Medical Sciences, Yazd, Iran;; 5 Department of Life Science Engineering, Faculty of New Sciences and Technologies, University of Tehran, Tehran, Iran

**Keywords:** Drug delivery systems, Graphene oxide, Nanoparticle, Quercetin

## Abstract

**Background:**

Enhancing the therapeutic profile of hydrophobic drugs using the development of biocompatible drug delivery systems is an urgent need. Many types of research have been conducted on graphene derivatives owing to their unique characteristics.

**Methods:**

In this survey, QUER, a natural medicine, was loaded on carboxylated GO, and cytotoxicity assay and the uptake of QUER into prostate cancer cells (PC3) were evaluated.

**Results:**

The release behavior of QUER was temperature- and pH-sensitive. Although QUER was loaded with high efficiency, the released rate was low (23.25% at pH 5.5 and 42 °C). The toxicity and intensity of fluorescence in the FREE QUER were higher than the loaded form.

**Conclusion:**

High-capacity loading and controlled release of GO QUER can be recognized as a proper candidate in treating cancer.

## INTRODUCTION

Development of biocompatible drug delivery systems for enhancing the therapeutic profile of hydrophobic drugs is an urgent demand. Nanomaterials can increase the efficacy of hydrophobic agents through enhancing drug solubility and cellular uptake and reducing systemic toxicity. The most important features of a proper drug delivery system are high drug loading capacity and controlled and localized drug release near cancerous cells^[1-3]^. There is a remarkable attention toward the usage of graphene derivatives in biomedical applications owing to their unique properties. However, aggregation of graphene particles in polar solvents has restricted its biomedical applications^[4]^. 

Unlike graphene, GO has shown more solubility as well as more reactive sites for chemical interactions due to the presence of functionalized groups (such as hydroxyl, epoxide, and carboxyl, on its surface^[5,6]^), which can be lead to more application in gene transfection, biosensing, molecular imaging, and drug delivery^[7,8]^. Furthermore, large surface area, varied surface chemistry, and favorable dispersion in polar solution have presented this graphane as an effective drug carrier compared to other carbon nanomaterials^[7,9]^. 

The cytotoxicity of GO with and without doxorubicin was assayed on human multiple myeloma cells, presenting the inhibition of cell proliferation by GO/doxorubicin^[10]^. One study has shown greater release of curcumin from PEGylated GO in the basic environment than acidic one^[11]^. Another study used polyvinylpyrrolidone-functionalized GO for the co-delivery of QUER and gefitinib toward ovarian cancer cells^ [12]^.

In the current study, the cytotoxicity effect and cellular uptake rate of FREE QUER and GO QUER in PC3 cell line were investigated.

## MATERIALS AND METHODS

**Materials**

QUER (purity >95%), MTT, dimethyl sulfoxide, dialysis bag (MW¼ 12 kDa), and PBS tablets were obtained from Sigma-Aldrich (St. Louis, MO, USA). DIL Stain (1,1′-dioctadecyl-3,3,3′,3′-tetramethylindo carbocyanine perchlorate) and DAPI were purchased from Thermo Fisher Scientific (Waltham, MA, USA). GO was produced from GrapheneX (Sydney, Australia). 

**Morphological assessment**

Scanning electron microscope (Model EM3200, KYKY, China) and Brookhaven Corp Instruments (Holtsville, NY, USA) were used to evaluate the structure and zeta potential of GO. For capturing the images, first, 5 µl of suspension was poured on a glass plate to produce a thin layer of film. Then the sample was coated with a gold layer, and the images were recorded by a scanning electron microscope (model EM3200, KYKY, China).

**Carboxylation of GO**

GO carboxylation was conducted by the following stages: sonicating GO (2 mg/ml) for one hour, adding 72 mg of NaOH and stirring at room temperature for four hours, adding 0.4 ml of HCl (37% v/v), and removing the salts with deionized water by centrifuging at 4000 ×g for 10 min. 

**Drug loading on GO**

GO (1 mg/ml) was mixed with 0.5 mg/ml of QUER (dissolved in ethanol), followed by stirring at room temperature overnight. After removing unbounded drugs via centrifugation (15000 ×g for 10 min), the concentration of QUER was evaluated by a UV–Vis spectrophotometer (Epoch Box 998 America) at 470 nm. Finally, EE% was assessed by the following equation: 

EE% = loaded drug on GO (mg ml^-1^)/total drug (mg ml^-1^) × 100

**Drug release assay**

The release amount of QUER from GO was conducted using a 12 kDa cut-off dialysis tube immersing in PBS (pHs 7.4 and 5.5; at 37 °C and 42 °C), which was stirred for 72 h. Subsequently, 0.5 ml of dialysis solution was gathered at different time intervals to measure its absorbance via the UV–Vis spectrophotometer. The solution was then substituted with 0.5 ml of fresh PBS. In the end, the percentage of release was assessed based on the total drug-loaded concentration. 

**Cell culture assay**

The cell line of human prostate cancer (PC3) was purchased from the Pasteur Institute of Iran (Tehran). Standard conditions for culturing these cells were comprised of DMEM medium, 10% fetal bovine serum, and penicillin–streptomycin, all from Gibco (Grand Island, USA) at 37 °C and 5% CO_2_.

***In vitro*** **evaluation of cellular uptake**

Distribution of the FREE QUER and GO QUER in the cells was evaluated using the intensity of fluorescence, which was captured by fluorescence microscopy (Olympus, Japan). Following the culture of the cells in a six-well plate (1.5 × 10^5^ per well), incubation with the FREE and GO QUER was conducted for three hours. Then PBS (pH 7.4) and 95% ethanol solution were used for washing and fixing, respectively. After staining the cells with DAPI (1 mg/ml), images were finally recorded using fluorescence microscopy. 

**Cytotoxicity assay**

IC_50_ values of both FREE and GO QUER were measured by MTT assay. After 48-h incubation of each well with both QUER, the content of wells was removed following the incubation of each well with 90 µl of medium and 10 µl of MTT (5 mg/ml) for three hours. Dimethyl sulfoxide was used to dissolve the formazan crystals. Ultimately, the absorbance of samples at 570 nm was evaluated by an EPOCH spectrophotometer (Bio-Tek, Winooski, USA). 

**Statistical analysis**

All data were expressed as the mean ± SD, which was analyzed by GraphPad Prism version 6 (GraphPad, San Diego, CA). Also, one-way analysis of variance (ANOVA) and Tukey's multiple comparisons test were used to measure statistical difference, *p* value < 0.05. 

## RESULTS AND DISCUSSION

**Characterization of compositions**

**Fig. 1:**
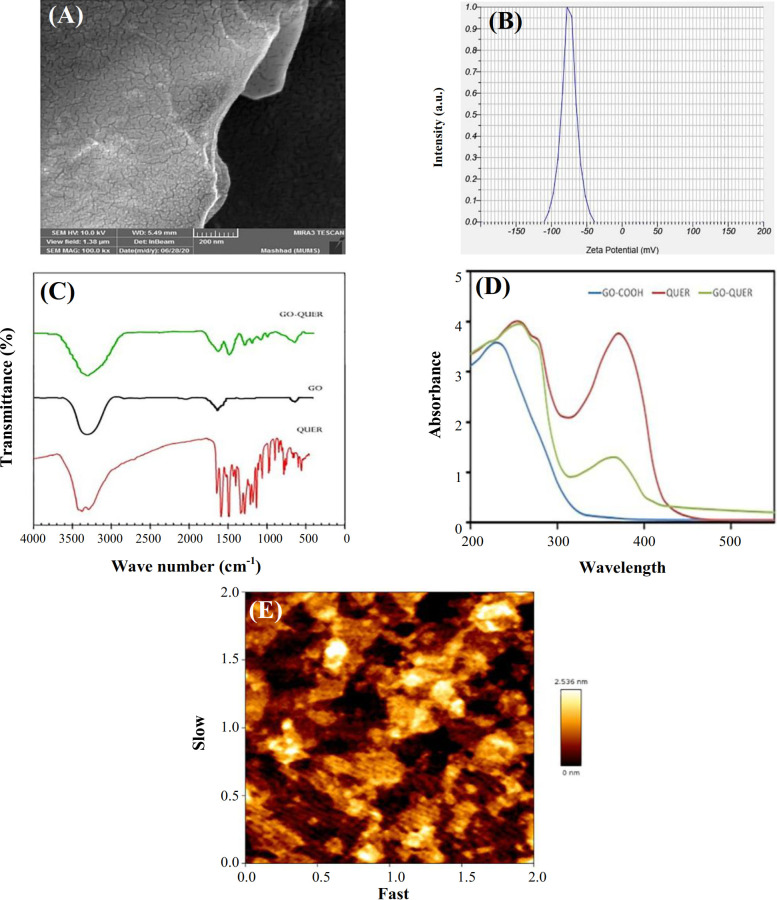
(A) GO scanning electron microscope images, (B) GO zeta potential, (C) FTIR spectrum of GO, GO QUER, and QUER, (D) UV–Vis spectrum of GO, GO QUER, QUER, and (E) atomic force microscopy image of GO, indicating less than 10 layers.

The sheet layer and broad surface of GO are depicted in Figure 1A^[13,14]^. Determination of zeta potential by dynamic light scattering, as an indicator of surface charge, has a main role in cellular interactions within the physiological system. In fact, attraction or repulsion between the particles was determined by the amount of zeta potential^[15]^. Greater stability has been found in the higher negative or positive value of zeta potential that particles like to disperse. In contrast, the lower value of zeta potential causes the aggregation of particles^[16,17]^. Figure 1B shows the negative zeta potential of GO (-75.0 mV) presenting the existence of COOH group in its surface. Figure 1C indicates the FTIR spectrum of nanomaterials. The OH band vibration in GO-COOH is represented as a wide band at 3301 cm^-1^, while its functional group (C = O) is displayed at 1631 cm^-1[18]^. The hydroxyl and carbonyl groups of QUER have displayed peaks of about 3294 and 1671 cm^-1^, respectively. Also, cyclobenzene peaks appeared at 1614, 1513, and 1457 cm^-1^. The GO QUER represents the GO, functional groups (3301 and 1631 cm^-1^), as well as the QUER-related peaks (3300, 1620 and 1500 cm^-1^), which provide successful loading of QUER on the GO. The UV–Vis spectra of FREE QUER, GO, GO QUER are demonstrated in Figure 1D. The GO spectrum is characterized by a single peak at 230 nm, which is attributed to the molecular transfer of π → π associated with C-C aromatic rings^[19]^. As the maximum FREE QUER absorption is shown at 260 and 370 nm, in the loaded form, in addition to the red shift (260 nm), the 370 nm band is also observed^[11,20]^. Based on the atomic force microscopy images of GO (Fig. 1E), the dimensions of the GO plates are about 0.4 µm, and the thickness of the plates is about 3 nm. As the thickness of each layer of GO is about 0.2-1.1 nm^[21]^, this thickness indicates each GO plate contains 3-10 layers of GO.

**Fig. 2:**
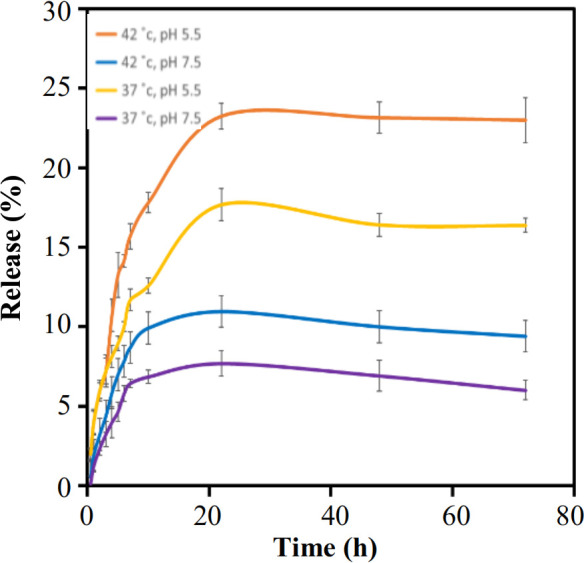
QUER drug release profile from GO QUER.

**Loading assay**

One of the main goals in the drug delivery systems is to obtain the high concentrations of loaded drug. The role of pH for achieving maximum loading of drugs on the GO-COOH is undeniable. For this purpose, the pH of drugs and GO-COOH (5.5-6) is needed to be similar, otherwise the accumulation of GO-COOH leads to low %EE; 84.5% of QUER was successfully loaded on the GO-COOH.

**Drug release assay**

Cancer cells have generally lower pH and higher temperature than normal cells^[22]^. *In vitro*, some important factors, such as pH, buffer temperature around nanoparticles, and GO membrane structure, affect drug release rate. In the present study, pH 7.5 and temperature of 37 °C were considered as a physiological condition, and pH 5.5 and temperature of 42 °C were regarded for cancer cells. The results revealed that the designed GO is sensitive to temperature and pH and results in better delivery of anticancer drugs to tumor cells, which in turn reduces side effects on normal cells. Based on Figure 2, QUER had the highest release rate at pH 5.5 and 23.25% at 42 °C. However, the lowest release rate was related to physiological conditions, 7.69. Our result was comparable to those of studies conducted by Pourjavadi *et al.*^[17]^, Malekmohammadi *et al.*^[23]^,, and Omidi *et al.*^[24]^. The low release rate of QUER was linked to its specific structure ans its hydrophobicity, which causes a stronger bond to GO^[24]^.

**Cytotoxicity assay**

**Fig. 3:**
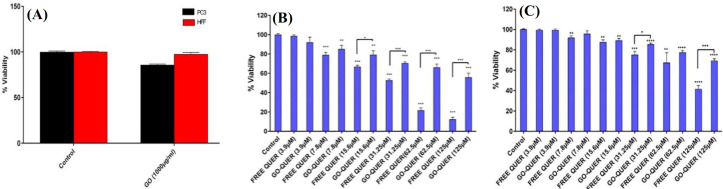
Cytotoxicity analysis of (A) FREE GO for PC3 after 48. Different concentrations of FREE QUER and GO QUER for PC3 (B) and HFF (C) after 48 h.

Although the use of chemotherapeutic agents is one of the main approaches to the treatment of cancer, drug resistance and its widespread effects on normal cells have limited effect. The study used a natural anticancer drug, QUER, which is significantly less toxic than commercially potent anticancer drugs, inexpensive, and readily available. MTT assay was performed to evaluate the inhibitory effects of different forms of drugs on PC3 cell line. First, the cytotoxicity of drug-free GO on PC3 cells was investigated. The results showed that GO at a concentration of 1,000 µg/ml had very minor toxicity, while this compound at the dose used in the analysis (200 µg/ml) had no toxicity and no effect on the survival rate of PC3 cells. Following cytotoxicity experiments, the effects of QUER on free and loaded forms were investigated. The results reflected that treatment with free and loaded drugs inhibited the growth of PC3 cells. As illustrated in Figure 3, the free form of the drug is more toxic than the loaded form. 

One of the main reasons for the use of nanocarriers in drug delivery is the loading of large amounts of drugs and, at the same time, their slow release to reduce the side effects of chemotherapy drugs. Based on the results, the IC_50_ of QUER in the PC3 cell line was 35.82 ± 2.84 µM, while in the loaded form (GO-QUER) increased (82.35 ± 4.55 µM). Also, The IC_50_ of QUER and GO-QUER in the HFF cell line was 91.10 ± 7.20 and 105 ± 6.17µM, respectively. These result showed that FREE QUER is more toxic than loaded drug, due to the strong binding of QUER to graphene oxide, which reduces its tendency to release. In all treatments, HFF cells, as a normal cell line, have higher IC_50_ values than cancer cells, indicating less toxicity in normal cells.

**Fig. 4:**
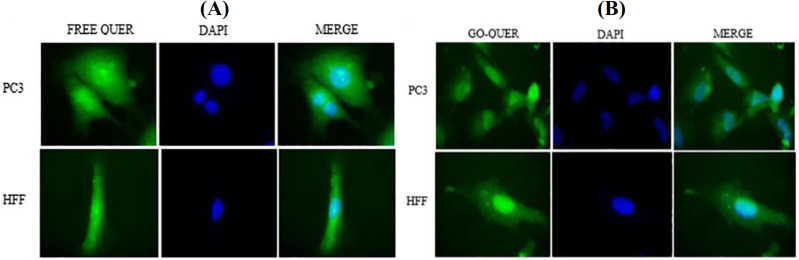
Cellular uptake images of PC3 cells, incubated with FREE QUER (A) and GO QUER (B) for 180 min. DAPI (blue) was used for nucleus staining.

**Cellular uptake assay**

Cellular uptake of FREE and GO QUER in PC3 cell line is depicted in Figure 4. GO and the nuclei of the cancer cells were stained with DAPI dye and DIL, DiIC18, respectively. These images show the successful delivery of GO and drug-loaded GOs into the cells. QUER emitted red fluorescence. It means that its intensity was higher in the free form of QUER compared to the loaded form because of the quenching property of GO^[25]^. Figure 4 represents lower fluorescence in the HFF cell line compared to PC3 cell line, indicating the lower entry of GO into normal cells. The result of Wu and co-workers^[10]^ suggested a similar result of the cellular uptake of free curcumin and curcumin-loaded lipid-polymer hybrid nano-particles. In the current study, cytotoxicity and cellular uptake of FREE and GO QUER in the PC3 cell line have been studied. While the loading efficiency of QUER on GO was high, the special structure of QUER reduced release rate. Based on the result of release, GO QUER was sensitive to pH and temperature, resulting in the differentiation of normal and cancerous cells. The fewer toxicity of GO QUER rather than FREE QUER displays localized and controlled release, which in turn leads to lesser side effect on normal cells. The effectiveness of GO QUER needs more release of QUER; therefore, photothermal and photodynamic therapy are suggested. 

GO was selected as a suitable nanoparticle owing to its bipolar properties and broad surface. While the release rate of QUER was low, its loading efficiency was more than 80%. The release profile was influenced by temperature and pH, which can differentiate normal and cancer cells. The higher IC_50_ values of the drug loaded on GO relative to the free form presented controlled release and more capability of drug loading, resulting in fewer side effects on normal cells. 

## DECLARATIONS

### Acknowledgments

The authors thank the Biochemistry Department at Shahid Sadoughi University of Medical Sciences and Medical Nanotechnology and Tissue Engineering Research Center, both in Yazd, Iran for their financial support.

### Ethical statement

Not applicable. 

### Data availability

The analyzed data sets generated during the study are available from the corresponding author on reasonable request. 

### Author contributions

FY: prepared the proposal, wrote the manuscript, and performed study analysis; NSHM: wrote the manuscript and performed study analysis; AM wrote the manuscript and performed study analysis; FH conceived the idea and supervised the study. All authors have read and approved the final version of the manuscript.

### Conflict of interest

None declared.

### Funding/support

The present study has financially supported by the Biochemistry Department at Shahid Sadoughi University of Medical Sciences and Medical Nanotechnology and Tissue Engineering Research Center, both in Yazd, Iran.
